# Electroacupuncture Alleviates Paclitaxel-Induced Peripheral Neuropathic Pain in Rats via Suppressing TLR4 Signaling and TRPV1 Upregulation in Sensory Neurons

**DOI:** 10.3390/ijms20235917

**Published:** 2019-11-25

**Authors:** Yuanyuan Li, Chengyu Yin, Xiaojie Li, Boyu Liu, Jie Wang, Xiaoli Zheng, Xiaomei Shao, Yi Liang, Junying Du, Jianqiao Fang, Boyi Liu

**Affiliations:** Key Laboratory of Acupuncture and Neurology of Zhejiang Province, Department of Neurobiology and Acupuncture Research, The Third Clinical Medical College, Zhejiang Chinese Medical University, Hangzhou 310053, Chinachengyu.yin@hotmail.com (C.Y.); 18300670930@163.com (X.L.); lby_0731@163.com (B.L.); bemyhalo@163.com (J.W.); zxl861260876@163.com (X.Z.); 13185097375@163.com (X.S.); dujunying0706@163.com (J.D.)

**Keywords:** Paclitaxel, acupuncture, dorsal root ganglion, peripheral neuropathy, TRPV1, glial cell, TLR4

## Abstract

Paclitaxel-induced peripheral neuropathy is a common adverse effect during paclitaxel treatment resulting in sensory abnormalities and neuropathic pain during chemotherapy and in cancer survivors. Conventional therapies are usually ineffective and possess adverse effects. Here, we examined the effects of electroacupuncture (EA) on a rat model of paclitaxel-induced neuropathic pain and related mechanisms. EA robustly and persistently alleviated paclitaxel-induced pain hypersensitivities. Mechanistically, TLR4 (Toll-Like Receptor 4) and downstream signaling MyD88 (Myeloid Differentiation Primary Response 88) and TRPV1 (Transient Receptor Potential Vallinoid 1) were upregulated in dorsal root ganglion (DRGs) of paclitaxel-treated rats, whereas EA reduced their overexpression. Ca^2+^ imaging further indicated that TRPV1 channel activity was enhanced in DRG neurons of paclitaxel-treated rats whereas EA suppressed the enhanced TRPV1 channel activity. Pharmacological blocking of TRPV1 mimics the analgesic effects of EA on the pain hypersensitivities, whereas capsaicin reversed EA’s effect. Spinal astrocytes and microglia were activated in paclitaxel-treated rats, whereas EA reduced the activation. These results demonstrated that EA alleviates paclitaxel-induced peripheral neuropathic pain via mechanisms possibly involving suppressing TLR4 signaling and TRPV1 upregulation in DRG neurons, which further result in reduced spinal glia activation. Our work supports EA as a potential alternative therapy for paclitaxel-induced neuropathic pain.

## 1. Introduction

Paclitaxel is among the frontline chemotherapeutic reagents for treating solid tumors, including ovarian, breast, non-small cell lung cancer, and head and neck cancer, etc. [1,2]. Paclitaxel acts to stabilize the assembly of intracellular microtubules, which results in cell death via interfering with microtubule functions required for cell division [3]. Its major side effects include chemotherapy-induced peripheral neuropathy (CIPN), which occurred in almost all patients in a dose-dependent manner [4,5]. The peripheral neuropathy causes neuropathic pain symptoms, which are characterized with burning pain, tingling, numbness, and allodynia in the hands and feet of patients [6,7]. The sensory abnormalities and pain symptoms often become chronic, which is refractory to treatment and significantly affects the life quality of the patients [8]. At present, conventional treatments for paclitaxel-induced peripheral neuropathy include nonsteroidal anti-inflammatory drugs, opioids, corticosteroids, and antidepressants, etc. [9] Unfortunately, these treatment options usually have limited therapeutic effects and cause many adverse effects [9,10]. Thus, paclitaxel-induced peripheral neuropathy still remains a pressing clinical symptom.

TRPV1 (Transient Receptor Potential Vallinoid 1) channel is a nonselective cation channel mainly expressed in nociceptive primary sensory neurons [11]. It is activated by a variety of noxious stimuli, such as capsaicin, protons, noxious heat, and some endogenous-inflammation-associated lipid metabolites, and therefore generates pain signal upon activation [12,13,14]. TRPV1 expression is upregulated in dorsal root ganglion (DRG) neurons of rats treated with paclitaxel, whereas TRPV1 antagonists significantly reduced pain hypersensitivities, suggesting that TRPV1 makes major contributions to paclitaxel-induced neuropathic pain symptoms [15,16,17,18].

TLR4 (Toll-Like Receptor 4) plays an important role in mediating chronic pain [19,20]. The expression of TLR4 and its downstream signaling molecule MyD88 (Myeloid Differentiation Primary Response 88) was found to be upregulated in DRG neurons of rats treated with paclitaxel [15,21]. Pharmacological blocking or genetic deletion of TLR4 or MyD88 attenuates paclitaxel-induced neuropathic pain [21,22]. TLR4 and TRPV1 are co-expressed in DRG neurons [15]. TRPV1 expression and activity in DRG neurons can be enhanced by TLR4 in chronic inflammation and paclitaxel-induced neuropathic pain conditions [15,23]. Therefore, paclitaxel may engage with TLR4 and its downstream signaling to promote the expression and activity of TRPV1 channel in peripheral sensory neurons, resulting in augmented nociceptive signals that contribute to neuropathic pain.

Acupuncture is a traditional and effective method for pain relief with few side effects. Acupuncture, in combination with reflexology, can ameliorate the symptoms of CIPN in patients with breast cancer [24]. More recently, a phase IIA clinical trial of acupuncture demonstrated that acupuncture is safe and shows effectiveness in improving the symptoms of patients with CIPN during chemotherapy [25]. Electroacupuncture (EA) combines traditional manual acupuncture with modern electrotherapy and has become a convenient method for pain relief in wide varieties of pain conditions [26,27]. It has demonstrated that EA can ameliorate neuropathic pain conditions of paclitaxel-treated rats [28,29]. However, the underlying mechanisms of EA’s therapeutic effects are still not fully understood. Mechanistic studies of EA’s therapeutic effects may further promote its application in clinical management of paclitaxel-induced neuropathic pain. Our previous studies demonstrated that EA can reduce TRPV1 overexpression in DRG neurons to ameliorate chronic pain [30,31]. Therefore, in the present study, we aim to explore whether EA may alleviate paclitaxel-induced neuropathic pain via suppressing TLR4 signaling and TRPV1 upregulation in DRG neurons.

## 2. Results

### 2.1. EA Significantly Alleviated both Thermal and Mechanical Hypersensitivities in a Rat Model of Paclitaxel-Induced Peripheral Neuropathic Pain

**Figure 1:**
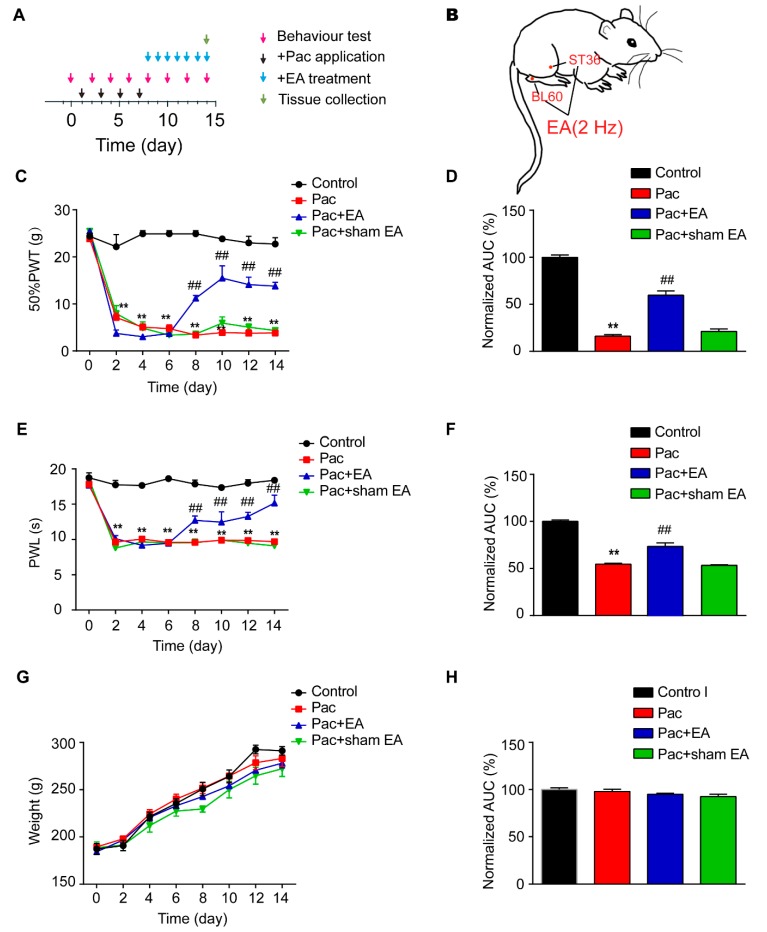
Electroacupuncture (EA) attenuates both mechanical allodynia and thermal hyperalgesia in a rat model of paclitaxel-induced peripheral neuropathic pain. (**A**) Experimental protocol for the establishment of the rat model of paclitaxel-induced peripheral neuropathic pain and EA/sham EA treatment. (**B**) A schematic picture illustrating the location of ST36 (5 mm lateral to the anterior tubercule of the tibia) and BL60 (at the ankle joint level and between the tip of the external malleolus and calcaneus) acupoints in the rat. (**C**) Time course effect of the repeated 2 Hz EA treatment on mechanical allodynia of paclitaxel-treated rats. (**D**) Normalized area under the curve (AUC) analysis of Figure 1C. (**E**) Time course effect of the repeated 2 Hz EA treatment on thermal hyperalgesia of paclitaxel-treated rats. (**F**) Normalized AUC analysis of Figure 1E. (**G**) Time course effect of paclitaxel and EA treatment on body weight of rats. (**H**) Normalized AUC analysis of Figure 1G. AUCs were all normalized to the corresponding control group. ** *p* < 0.01 vs. control group. ^##^ *p* < 0.01 vs. Pac + sham EA group. *n* = 5 rats/group. One-way or two-way ANOVA followed by Tukey post hoc test was used for statistical analysis.

We first established the rat model of paclitaxel-induced peripheral neuropathic pain via multiple paclitaxel injections according to protocols reported before [32,33] (Figure 1A). Administration of cumulative dosages of 24 mg/kg paclitaxel (4 × 6 mg/kg, 2 days apart, intraperitoneal (i.p.) elicited robust and persistent reduction in the 50% paw withdraw threshold (PWT) in rats (Pac group), a sign of mechanical allodynia, compared with vehicle-treated rats (Control group). The mechanical allodynia lasted till the end of our observation time frame (day 14). In addition, paclitaxel-injected rats also developed obvious signs of thermal hyperalgesia, manifested by a significant reduction of paw withdraw latency (PWL) in Hargreaves test, as compared with the vehicle-treated rats (control group). The thermal hyperalgesia persisted until the end of the observation time frame as well (day 14) (Figure 1C). These results were consistent with previous reports, indicating the successful establishment of paclitaxel-induced peripheral neuropathic pain in rats.

We then applied 2 Hz EA on bilateral ST36 and BL60 acupoints located on the hind limbs of the rat (Figure 1B). These two acupoints were frequently used in our previous studies and showed reliable analgesic effects on hind limbs upon EA stimulation [34]. Sham EA, with needles inserted into ST36 and BL60 acupoints but with no current stimulation, was used as a negative control (Sham EA group). EA was applied for 30 min on a daily basis starting on day 8, one day after the last paclitaxel injection. Sham EA produced no anti-allodynic effect compared with paclitaxel-treated rats (Pac group) (Figure 1C), whereas EA produced robust and persistent anti-allodynic effects until the end of the observation time frame compared with the Pac + sham EA group (Figure 1C). Area under the curve (AUC) analysis further demonstrated an overall anti-allodynic effect of EA treatment on paclitaxel-treated rats (Figure 1D). In addition, EA produced persistent relief of thermal hyperalgesia of paclitaxel-treated rats, whereas sham EA was not effective (Figure 1E). AUC analysis further indicated an overall effect of EA on thermal hyperalgesia of paclitaxel-treated rats (Figure 1F). In addition, paclitaxel treatment did not affect the body weight of rats compared with the vehicle group and a repeated EA treatment had no effect on the body weight either (Figure 1G,H).

### 2.2. EA Reduced the Overexpression of TLR4, MyD88, and TRPV1 in DRGs of Paclitaxel-Treated Rats

**Figure 2:**
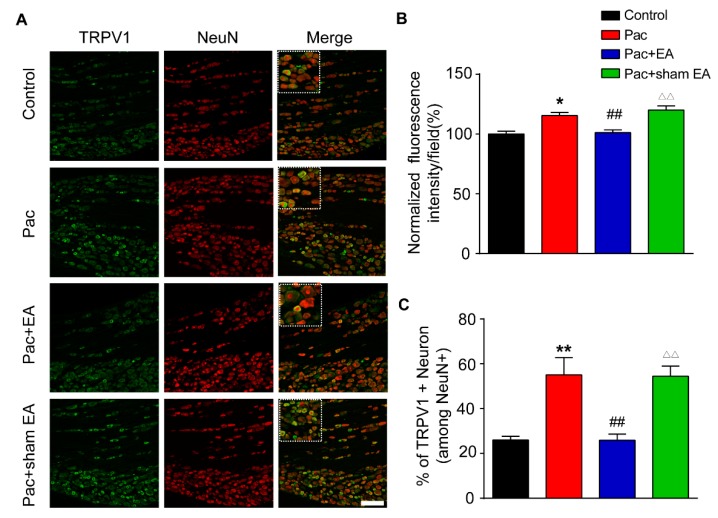
EA reduced the upregulation of TRPV1 (Transient Receptor Potential Vallinoid 1) channel expression in dorsal root ganglion (DRG) neurons from paclitaxel-treated rats. (**A**) Representative immunofluorescence images indicating TRPV1 antibody staining of DRG neurons from the control, Pac, Pac + EA, and Pac + sham EA groups. Areas staining positive for TRPV1 are shown in green. Slices were co-stained with NeuN antibody (in red) to identify all DRG neurons. Scale bar indicates 100 μm. (**B**) Summary of the normalized % increase in fluorescence intensity of TRPV1 immunostaining in each observation field. The value of each group was normalized to that of the control group. (**C**) Summary of the % of TRPV1 positively stained neurons (TRPV1^+^) from each observation field. The total number of DRG neurons per observation field was deduced from positive NeuN (NeuN^+^) staining. *n* = 5 rats/group. * *p* < 0.05, **^,^^△△^ *p* < 0.01 vs. control group. ^##^ *p* < 0.01 vs. Pac + sham EA group. One-way ANOVA followed by Tukey post hoc test was used for statistical analysis.

**Figure 3:**
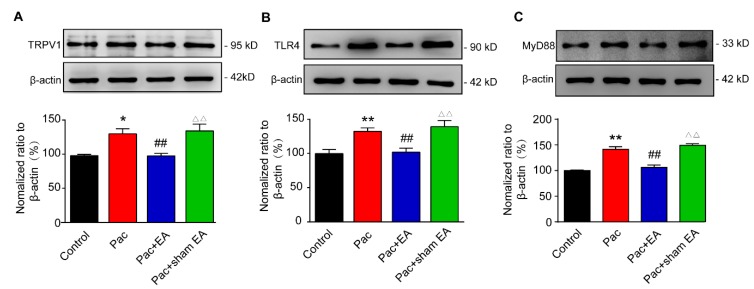
EA attenuates the upregulation of TLR4 (Toll-Like Receptor 4), MyD88 (Myeloid Differentiation Primary Response 88), and TRPV1 protein expression in DRGs of paclitaxel-treated rats. The determination of TRPV1 (**A**), TLR4 (**B**) and MyD88 (**C**) protein expression by Western blot in rat DRGs: Upper panel shows representative images of TRPV1, TLR4, and MyD88 and of β-actin protein expression from the control, Pac, Pac + EA, and Pac + sham EA groups. Lower panel shows the summarized TRPV1, TLR4, and MyD88 protein expression normalized to β-actin. * *p* < 0.05 and **^,^^△△^ *p* < 0.01 vs. control group. ^##^ *p* < 0.01 vs. Pac + sham EA group. *n* = 5 rats/group. One-way ANOVA followed by Tukey post hoc test was used for statistical analysis.

We then investigated the mechanisms underlying EA-induced analgesic effects on paclitaxel-treated rats. It is well established that TRPV1 channel expression is increased in DRGs upon paclitaxel treatment and plays a critical role in mediating paclitaxel-induced peripheral neuropathic pain [15,16]. Our immunofluorescence study revealed that the percentage of TRPV1 immune positive (TRPV1^+^) DRG neurons among all DRG neurons (stained with NeuN) and TRPV1 immunofluorescent staining intensity were both significantly increased in the paclitaxel-treated group (Figure 2A–C). Repeated EA treatment significantly reduced the overexpression of TRPV1 induced by paclitaxel treatment (Figure 2A–C). In contrast, sham EA had no effect on TRPV1 overexpression (Figure 2A–C). We further examined the expression of TRPV1 in DRGs by Western blotting. Western blotting revealed that TRPV1 expression was significantly increased in the L4–6 DRGs of paclitaxel-treated rats (Figure 3A). Repeated EA treatment significantly reduced TRPV1 overexpression in L4–6 DRGs, whereas sham EA had no effect (Figure 3A).

TLR4 and its downstream signaling molecule MyD88 are both increased in DRGs of paclitaxel-treated rats and are involved in paclitaxel-induced peripheral neuropathic pain [15,21]. Evidence suggests that TLR4 promotes TRPV1 overexpression in DRG neurons during paclitaxel-induced peripheral neuropathy and inflammation [15,23]. Then, we continued to examine the effects of EA on the expression of TLR4 and MyD88 in DRGs of paclitaxel-treated rats. Consistent with previous reports, we found that the expression of TLR4 and MyD88 were both significantly upregulated in L4–6 DRGs of paclitaxel-treated rats compared with vehicle-treated rats by Western blotting (Figure 3B,C). Moreover, EA significantly reduced the upregulation of TLR4 and MyD88 expression in DRGs of paclitaxel-treated rats, whereas sham EA had no such effect (Figure 3B,C).

### 2.3. TRPV1 Channel Functional Activity is Enhanced in DRG Neurons of Paclitaxel-Treated Rats, and EA Eliminated the Enhancement of TRPV1 Activity by Paclitaxel

**Figure 4:**
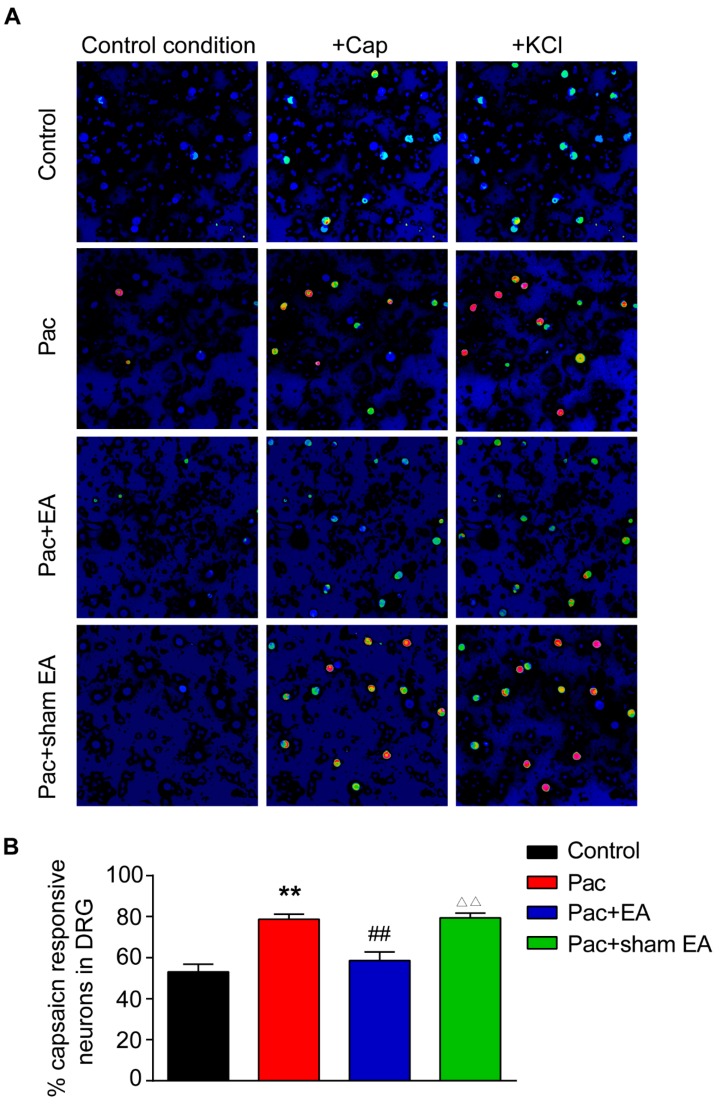
Paclitaxel treatment increased the percentage of capsaicin-positive responding DRG neurons, and EA attenuates the effect of paclitaxel determined by Ca^2+^ imaging. (**A**) Pseudo color images from Fura-2-based ratiometric imaging showing the Ca^2+^ responses in DRG neuron in response to TRPV1 specific agonist capsaicin (Cap, 100 nM) in the control, Pac, Pac + EA, and Pac + sham EA groups. KCl (40 mM) was applied at the end of each recording to determine all active DRG neurons. (**B**) Summarized % of capsaicin responding DRG neurons in each observation field from the control, Pac, Pac + EA, and Pac + sham EA groups. *n*  =  6 tests/group, and each group contains 150–200 neurons derived from 3–4 rats. **^,^^△△^ *p* < 0.01 vs. control group. ^##^ *p* < 0.01 vs. Pac + sham EA group. One-way ANOVA followed by Tukey post hoc test was used for statistical analysis.

Since TRPV1 channel expression is upregulated in DRG neurons by paclitaxel treatment, we then performed live cell Ca^2+^ imaging to monitor the functional activities of TRPV1 channel in acutely dissociated L4–6 DRG neurons from paclitaxel-treated rats. Capsaicin, the specific TRPV1 agonist, was used to probe TRPV1 channel activities. KCl (40 mM) was perfused at the end of the recording to monitor all live DRG neurons. We found that perfusion of capsaicin (100 nM) elicited more percentage of capsaicin-responding neurons from paclitaxel-treated rats compared with vehicle-treated rats (Figure 4A,B). Repeated EA treatment significantly reduced the increase in percentage of capsaicin responding neurons caused by paclitaxel, whereas sham EA had no obvious effect (Figure 4A,B).

**Figure 5:**
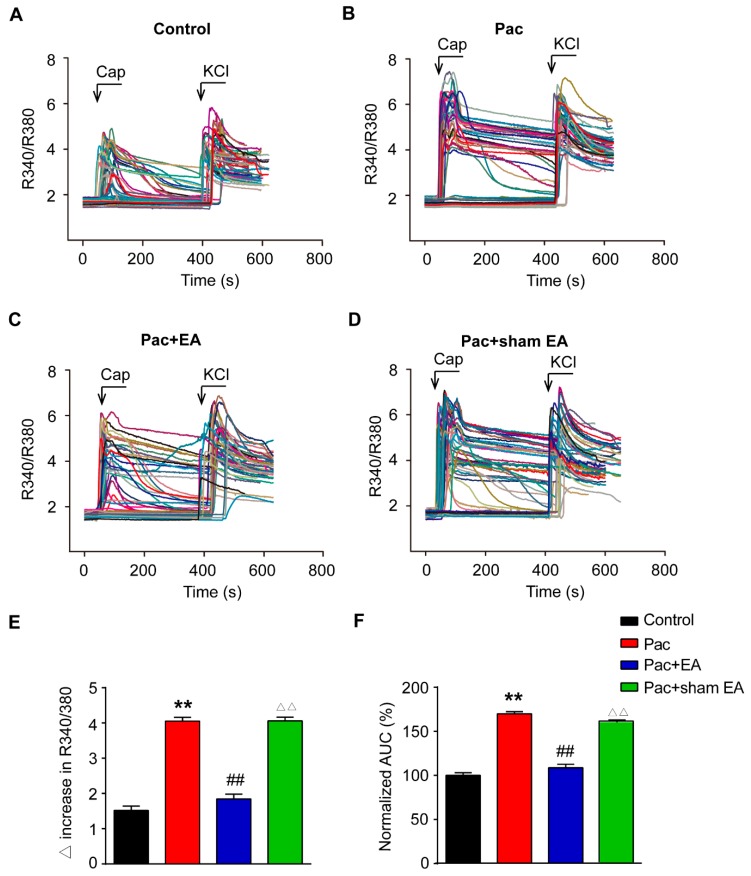
Paclitaxel treatment increased the amplitude of capsaicin-induced Ca^2+^ response in DRG neurons, and EA attenuated the effect of paclitaxel. Overlaid Ca^2+^ imaging traces of DRG neurons isolated from the Control (**A**), Pac (**B**), Pac + EA (**C**), and Pac + sham EA (**D**) groups of rats: DRG neurons were perfused with capsaicin (Caps, 100 nM), followed with KCl (40 mM). The arrows and bars indicate the time point and duration of drug application, respectively. (**E**) Summarized data showing the Δ increase in the peak 340/380 ratio before and after capsaicin application. (**F**) Summarized data showing the AUC of the Ca^2+^ imaging traces during capsaicin application. **^,^^△△^ *p* < 0.01 vs. control group. ^##^ *p* < 0.01 vs. Pac + sham EA group. *n*  =  6 tests/group, and each group contains 150–200 neurons derived from 3–4 rats. One-way ANOVA followed by Tukey post hoc test was used for statistical analysis.

Furthermore, we observed that the magnitude of capsaicin-induced Ca^2+^ response was significantly enhanced in DRG neurons isolated from paclitaxel-treated rats compared with vehicle-treated rats (Figure 5A,B). Detailed analysis of the peak amplitude of the Ca^2+^ traces as well as AUC of the Ca^2+^ traces further revealed that capsaicin produced more increase in these two parameters in DRG neurons from paclitaxel-treated rats compared with vehicle-treated rats (Figure 5E,F). Repeated EA treatment significantly reduced paclitaxel-induced increase in peak amplitude and AUC of the Ca^2+^ traces, whereas sham EA had no effect (Figure 5C–F). Therefore, Ca^2+^ imaging experiments indicated that the functional activity of the TRPV1 channel was significantly increased in DRG neurons from paclitaxel-treated rats whereas repeated EA treatment attenuated the upregulated TRPV1 channel functional activity in DRG neurons induced by paclitaxel.

### 2.4. Pharmacological Blocking of TRPV1 Mimics EA’s Therapeutic Effect in Reducing Pain Hypersensitivities of Paclitaxel-Treated Rats

**Figure 6:**
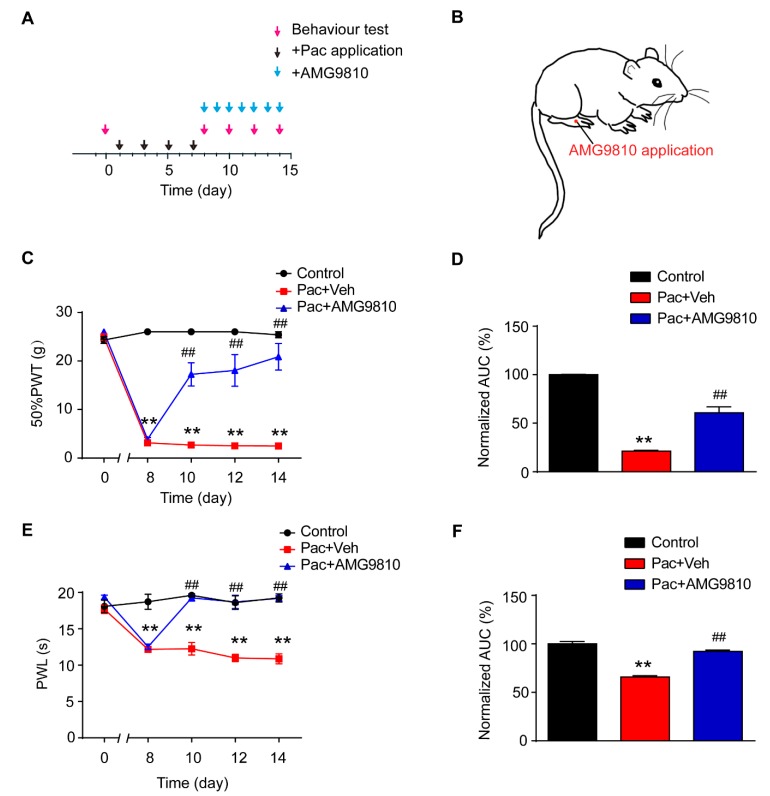
Pharmacological blocking of TRPV1 mimics EA’s therapeutic effects in reducing pain hypersensitivities of paclitaxel-treated rats. (**A**) Experimental protocol showing treatment schedule of AMG9810, the specific TRPV1 antagonist. (**B**) Schematic picture showing AMG9810 application site in the rat. (**C**) Time course effect of AMG9810 treatment on mechanical allodynia of paclitaxel-treated rats. (**D**) Normalized AUC analysis of Figure 6C. (**E**) Time course effect of AMG9810 treatment on thermal hyperalgesia of paclitaxel-treated rats. (**F**) Normalized AUC analysis of Figure 6E. *n* = 5 rats/group. ** *p* < 0.01 vs. control group. ^##^ *p* < 0.01 vs. Pac + Veh group. One-way or two-way ANOVA followed by Tukey post hoc test was used for statistical analysis.

We next examined the effects of AMG9810, the specific TRPV1 antagonist, on the pain hypersensitivities of paclitaxel-treated rats [35]. AMG9810 was applied daily to the right hind paw of paclitaxel-treated rat 45 min before nocifensive testing (Figure 6A,B). The dosage we used was derived from effective local dosage reported in our previous studies [36]. The rats that received AMG9810 treatment (Pac + AMG group) exhibited significantly attenuated mechanical allodynia as well as thermal hyperalgesia in the treated hind paw compared with rats treated with vehicle (Pac + Veh group). Analysis of AUC in Figure 6C,E showed an overall inhibition of the mechanical allodynia and thermal hyperalgesia by persistent AMG9810 treatment (Figure 6D,F).

### 2.5. EA’s Analgesic Effects on Paclitaxel-Induced Pain Hypersensitivities are Reversed by Application of TRV1 Agonist Capsaicin

**Figure 7:**
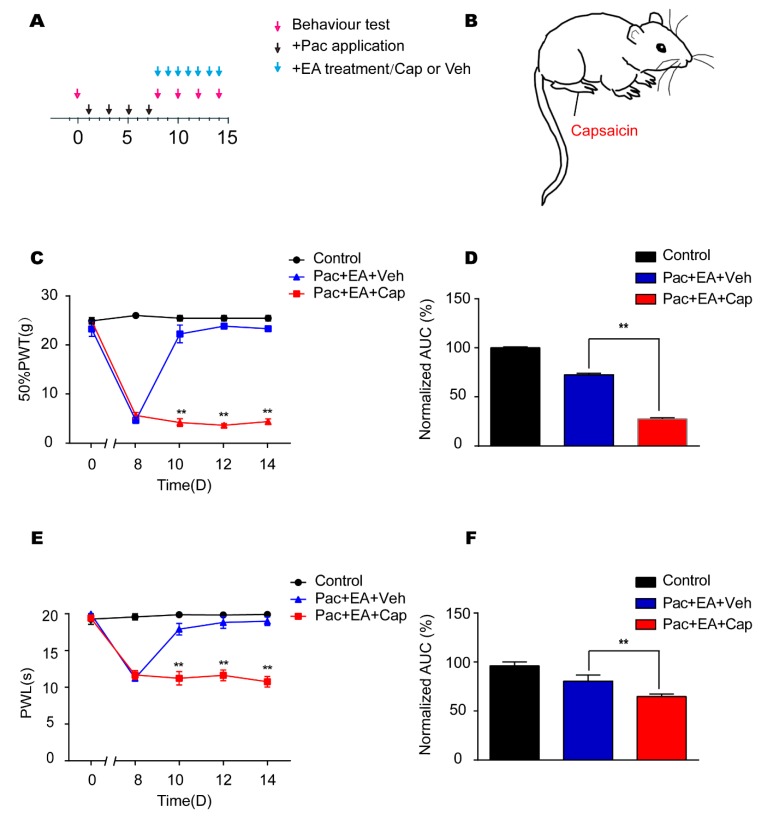
The TRPV1 agonist capsaicin reversed EA’s analgesic effects on paclitaxel-induced pain hypersensitivities. (**A**) Experimental protocol showing treatment schedule. (**B**) Schematic picture showing capsaicin injection site in the rat. (**C**) Time course effect of capsaicin treatment on EA’s anti-allodynic effect on paclitaxel-treated rats. (**D**) Normalized AUC analysis of Figure 7C. (**E**) Time course effect of capsaicin treatment on EA’s analgesic effect on thermal hyperalgesia of paclitaxel-treated rats. (**F**) Normalized AUC analysis of Figure 7E. *n* = 5 rats/group. ^**^ *p* < 0.01 vs. Pac + EA + Veh group. One-way or two-way ANOVA followed by Tukey post hoc test was used for statistical analysis.

To further confirm that EA’s analgesic effect on paclitaxel-treated rats was due to TRPV1 modulation, we tested the effect of TRPV1 agonist capsaicin on EA’s analgesic effect. Capsaicin was injected into the right hind paw of paclitaxel-treated rat on a daily basis after EA treatment (Figure 7A,B). Capsaicin significantly reversed the analgesic effects of EA on paclitaxel-induced mechanical allodynia (Figure 7C) and thermal hyperalgesia (Figure 7E) in rats. AUC analysis further demonstrated an overall reversal of the analgesic effects of EA on paclitaxel-treated rats by capsaicin (Figure 7D,F). These results further support the notion that EA’s analgesic effect on paclitaxel-induced pain hypersensitivities is mediated by TRPV1 modulation.

### 2.6. Repeated EA Treatments Reduce Astrocyte and Microglia Activations in the Spinal Cord Dorsal Horn of Paclitaxel-Treated Rats

**Figure 8:**
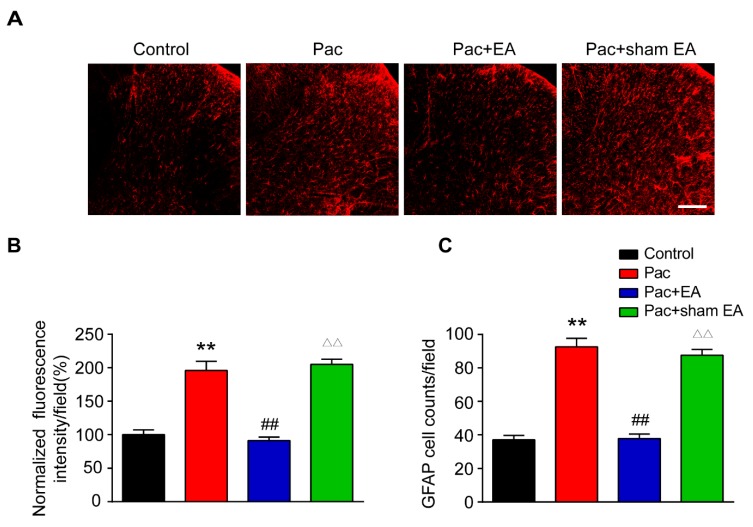
EA treatment attenuates astrocyte activation in the spinal cord dorsal horn (SCDH) of paclitaxel-treated rats. (**A**) Representative pictures of SCDH stained with astrocytic marker glial fibrillary acidic protein (GFAP) antibody showing astrocytes from the control, Pac, Pac + EA and Pac + sham EA groups. Scale bars = 50 μm. (**B**) Summary of the normalized fluorescence intensity (%) of GFAP staining in SCDH. (**C**) Summary of the number of astrocytes in each observation field in SCDH. *n* = 5 rats/group. **^,^^△△^ *p* < 0.01 vs. control group. ^##^ *p* < 0.01 vs. Pac + sham EA group. One-way ANOVA followed by Tukey post hoc test was used for statistical analysis.

**Figure 9:**
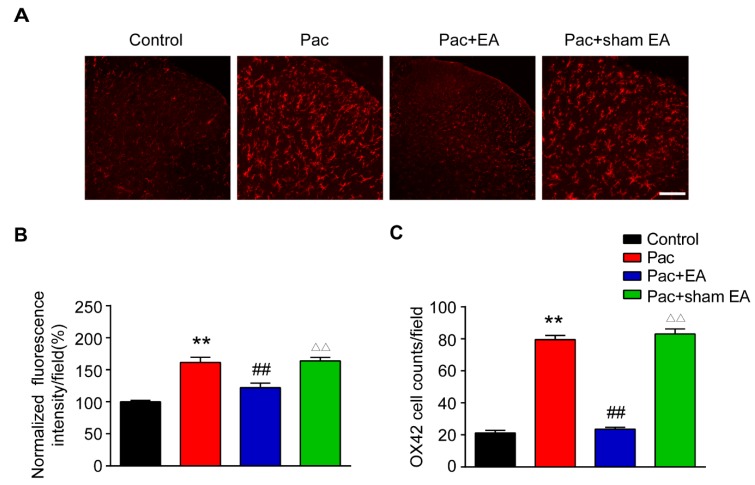
EA treatment attenuates microglia activation in SCDH of paclitaxel-treated rats. (**A**) Representative pictures of SCDH stained with microglial marker OX42 antibody showing microglia from the control, Pac, Pac + EA, and Pac + sham EA groups. Scale bars = 50 μm. (**B**) Summary of the normalized fluorescence intensity (%) of OX42 staining in SCDH. (**C**) Summary of the number of microglia in each observation field in SCDH. *n* = 5 rats/group. **^,^^△△^ *p* < 0.01 vs. control group. ^##^ *p* < 0.01 vs. Pac + sham EA group. One-way ANOVA followed by Tukey post hoc test was used for statistical analysis.

Glial cells in the spinal cord dorsal horn (SCDH) play critical roles in maintaining chronic pain [37,38]. Astrocytes and microglia are activated in SCDH of paclitaxel-induced peripheral neuropathic pain animals and are involved in pain mechanisms [39,40,41,42]. We then studied whether EA interferes with glial cell activities in SCDH. We examined the changes of immunostaining intensity of astrocytic marker glial fibrillary acidic protein (GFAP) and microglial marker OX42 in SCDH. Immunofluorescence showed that paclitaxel treatment significantly increased the staining intensity of GFAP and the number of GFAP positive cells in SCDH (Figure 8A–C). EA treatment significantly reduced the increased GFAP staining intensity and number of GFAP positive cells in SCDH of paclitaxel-treated rats (Figure 8A–C). In addition, paclitaxel treatment also significantly increased the staining intensity of OX42 and the number of OX42 positive cells in SCDH of paclitaxel-treated rats (Figure 9A–C). Similarly, EA treatment significantly attenuated the increased OX42 staining intensity and number of OX42 positive cells in SCDH of paclitaxel-treated rats (Figure 9A–C). These data demonstrated that paclitaxel-induced peripheral neuropathic pain is accompanied with significant astrocyte and microglia activation in SCDH and that EA effectively attenuates astrocyte and microglia activation in SCDH of paclitaxel-treated rats.

## 3. Discussion

In the present study, we established a rat model of paclitaxel-induced peripheral neuropathic pain and evaluated the therapeutic effects of EA on the pain hypersensitivities of paclitaxel-treated rats. We further investigated the potential mechanisms underlying EA’s analgesic effect. We found that TRPV1, TLR4, and its downstream signaling MyD88 were all upregulated in DRGs of paclitaxel-treated rats. EA treatment significantly reduced the overexpression of TLR4, MyD88, and TRPV1 in DRGs of paclitaxel-treated rats. By means of Ca^2+^ imaging, we found that TRPV1 channel functional activity is enhanced in DRG neurons of paclitaxel-treated rats whereas EA treatment significantly eliminated the enhancement of TRPV1 activity in DRG neurons of paclitaxel-treated rats. Pharmacological blocking of TRPV1 with specific antagonist AMG9810 mimics the effect of EA to reduce pain hypersensitivities, whereas capsaicin reversed the effect of EA. We further found that astrocytes and microglia were activated in SCDH of paclitaxel-treated rats whereas EA treatment reduced astrocyte and microglia activation in SCDH. These results suggest that EA alleviates paclitaxel-induced peripheral neuropathic pain via mechanisms possibly involving suppressing TLR4 signaling and TRPV1 channel upregulation in DRGs and spinal glial cell activation.

We found that TRPV1 expression is significantly upregulated in DRG neurons of paclitaxel-treated rats, a phenomenon consistent with previous publications [15,16]. Specific TRPV1 antagonists significantly reduced the pain hypersensitivities of paclitaxel-treated animals, demonstrating a critical role of TRPV1 in mediating the pain hypersensitivities [15,16]. Previous studies exclusively focused on the expression changes of TRPV1 in DRG neurons upon paclitaxel treatment [15,16,43]. In our study, we further evaluated the functional activities of TRPV1 channel by means of Ca^2+^ imaging in vitro. We found that TRPV1-mediated Ca^2+^ signals were significantly enhanced in DRG neurons acutely dissociated from paclitaxel-treated rats compared with control rats. This is by far the first study demonstrating the enhancement of functional activities of TRPV1 channel in DRG neurons from rats treated with paclitaxel, which further confirmed the upregulation of TRPV1 channel expression by paclitaxel. We further examined the effects of EA on TRPV1 channel expression and activities in DRG neurons from paclitaxel-treated rats. We found that EA significantly reduced TRPV1 overexpression as well as the enhanced channel activity in DRG neurons from paclitaxel-treated rats. Although some previous studies reported that EA can reduce TRPV1 channel overexpression, none have examined EA’s effect on TRPV1 channel functional activities. Therefore, our study provides the first evidence showing that EA can reduce both TRPV1 channel overexpression and channel functional activities in DRG neurons from paclitaxel-treated rats.

TLR4/MyD88 has been reported to be involved in paclitaxel-induced peripheral neuropathy in rats [21]. In that study, it is reported that TLR4 and MyD88 expression are both increased in DRGs of paclitaxel-treated rats. The TLR4 antagonist blocked the increased expression of both TLR4 and MyD88. Furthermore, blocking TLR4 or MyD88 both significantly reduced the behavioral hypersensitivities of paclitaxel-treated rats [15,21]. Therefore, TLR4/MyD88 signaling is involved in paclitaxel-induced peripheral neuropathy. Moreover, in another report, MyD88-conditional knockout mice by deleting MyD88 in nociceptive neurons ameliorated the pain responses of paclitaxel-treated rats [22]. Therefore, these data all suggest that TLR4/MyD88 is functionally related with paclitaxel-induced peripheral neuropathy.

The link between TLR4 and TRPV1 in pain conditions, including paclitaxel-induced peripheral neuropathic pain, has been well documented. It is reported that TLR4 and TRPV1 are co-expressed in DRG neurons [15]. More importantly, TRPV1 expression or channel activity in DRG neurons can be significantly enhanced by TLR4 under inflammation or paclitaxel-induced neuropathic pain conditions [15,23,44,45]. Furthermore, pharmacological blocking of TLR4 significantly reduced the overexpression of TRPV1 in DRG neurons and reduced the pain response of paclitaxel-treated rats [15]. These studies all suggested that TLR4 can enhance TRPV1 expression and channel activity in DRG neurons during paclitaxel-induced peripheral neuropathic pain.

In this study, we found that the expressions of TLR4 and MyD88 were both significantly increased in DRG neurons of paclitaxel-treated rats, a result consistent with previous findings [15,21]. More importantly, we found that EA treatment significantly reduced the overexpression of TLR4 and MyD88 in DRGs of paclitaxel-treated rats. This finding indicated that the effect of EA on TRPV1 may possibly be related with its effect on the upstream signaling of TLR4 and MyD88 in DRGs. It is well established that TLR4 signaling is largely mediated by MyD88, which in turn activates the NF-kappaB inhibitor alpha (NF-κB) and Mitogen-activated protein (MAP) kinase pathways, leading to production of inflammatory cytokines, including Interleukin-1 beta (IL-1β) and Tumor necrosis factor- alpha (TNF-α) [19,46]. These inflammatory cytokines are capable of enhancing TRPV1 expression in DRG neurons [47,48]. Therefore, it still remains to be investigated whether EA can further reduce NF-κB or MAP kinase pathways-induced inflammatory cytokine release in DRG neurons of paclitaxel-treated rats.

It is reported that paclitaxel-induced peripheral neuropathic pain is accompanied with astrocyte and microglia activation in SCDH. Pharmacological blocking of astrocytes or microglia attenuated paclitaxel-induced neuropathic pain, suggesting spinal glial cells contributed to pain mechanisms of paclitaxel-induced neuropathic pain [40,41,42]. Here, we found that astrocyte and microglia are both activated in SCDH of paclitaxel-treated rats, which is consistent with previous studies. More importantly, EA treatment significantly alleviated the astrocyte and microglia activation in SCDH of paclitaxel-treated rats. This result suggests that EA may alleviate paclitaxel-induced neuropathic pain via suppressing astrocyte and microglia activation in SCDH.

In the present study, we employed the sham EA group, in which acupoints ST36 and BL60 were subcutaneously inserted with needles but without electrical stimulation. This type of sham EA control can help to authenticate the therapeutic effects of electrical stimulation on pain response and is a widely accepted form of sham control for EA studies [34,49,50,51,52]. However, this type of sham control cannot confirm acupoint specificity in EA manipulation, which constitutes certain limitations. Therefore, more types of sham controls need to be employed to thoroughly evaluate the effects of both electrical stimulation and acupoint specificity in future EA studies. For instance, in addition to the sham EA we currently employed, another sham EA control could receive EA at a sham point 5 mm away from ST36 or BL60 after paclitaxel treatment, which can help to explain the acupoint specificity.

## 4. Materials and Methods

### 4.1. Animals

Male Sprague–Dawley (SD) rats (5–8 weeks, 180–220 g) were purchased from Shanghai Laboratory Animal Center, Chinese Academy of Sciences and housed in the Laboratory Animal Center of Zhejiang Chinese Medical University accredited by the Association for Assessment and Accreditation of Laboratory Animal Care (AAALAC). The rats were randomly allocated and were housed in a controlled environment (5 rats per cage on 12 h light–dark cycles with controlled temperature). Food and water were provided ad libitum. The rats were given a minimum of 1 week to adapt to new environment before the experiment. All experimental procedures were carried out in accordance with National Institutes of Health guidelines for the care and use of Laboratory animals (NIH Publications No. 8023, revised 1978) and approved by the Animal Ethics Committee of Zhejiang Chinese Medical University (ZSLL, 2017-183).

### 4.2. CIPN Rat Model Establishment

The rat CIPN model was established according to methods previously described [32,33]. Briefly, 6 mg/mL of stock pharmaceutical-grade paclitaxel (Hospira Australia Pty. Ltd., Australia) was diluted with sterile 0.9% saline to 1 mg/mL and given at a dosage of 2 mg/kg intraperitoneally (i.p.) every other day for a total of four injections (days 1, 3, 5, and 7). Control animals received the same volume of saline treatment. Rats were observed carefully for any abnormal behavioral changes every other day following the treatment.

### 4.3. EA Treatment

The procedure of EA was carried out according to our previous study [34]. Briefly, rats were loosely immobilized, and acupuncture needles of 0.25 mm in diameter were inserted at a depth of 5 mm into bilateral Zusanli (ST36, 5 mm lateral to the anterior tubercule of the tibia) and Kunlun (BL60, at the ankle joint level and between the tip of the external malleolus and calcaneus) acupoints. The needles were connected with HANS acupuncture point nerve stimulator (HANS-200A Huawei Co., Ltd., Beijing, China). The parameters were set as follows: 2 Hz, square wave current output (pulse width: 0.2 ms). Intensities ranging from 0.5 to 1.5 mA (increased by 0.5 mA every 10 min, for a total of 30 min) were delivered for a period of 30 min. For sham EA treatment, rats were inserted with needles subcutaneously (1 mm depth into ST36 and BL60) but without electrical stimulation. The treatment was conducted once daily for 7 consecutive days.

### 4.4. Mechanical Allodynia

Rats were placed in the test environment daily for 3 consecutive days before baseline test to habituate the test environment. Before the test, rats were individually placed in transparent Plexiglas chambers on an elevated mesh floor for 30 min to acclimate the test environment. The mechanical allodynia was determined using a series of *von* Frey filaments (UGO Basile, Italy) applied perpendicularly to the mid-plantar surface of the hind paws, with sufficient force to bend the filament slightly for 3–5 s according to methods we previously used [53]. An abrupt withdrawal of the paw, licking, or vigorously shaking in response to stimulation were considered pain-like responses. The threshold was determined using the up-down testing paradigm, and the 50% paw withdrawal threshold (PWT) was calculated by the nonparametric Dixon test [54,55]. A baseline test of PWT was done every day for 3 consecutive days before formal testing to acclimatize the rats and to ensure that there were no differences among groups.

### 4.5. Thermal Hyperalgesia

The Plantar Test Apparatus (Ugo Basile, Italy) was used to evaluate thermal hyperalgesia. Rats were habituated for 30 min before the test. A radiant light beam generated by a light bulb was directed into the right hind paw in order to determine the paw withdrawal latency (the time spent to remove the paw from the stimulus). A 20-s cutoff threshold was set to avoid excessive heating to cause injury. Significant decreases in paw withdrawal latency (PWL) were interpreted as heat hyperalgesia. Behavior tests were performed by an experimenter blinded to groupings.

### 4.6. Drug Administration

AMG9810, the TRPV1 specific antagonist (Tocris, USA) was prepared in Dimethylsulfoxide (DMSO) as a stock and diluted into PBS (1:1000). AMG9810 was applied via intraplantar injection (0.8 mg/25 μL) to the dorsal part of the ipsilateral hind paw 45 min before the test. The dosage of AMG9810 is derived from previous studies [36]. Rats of the sham group were administered with vehicle (0.1% DMSO in PBS) only. AMG9810 or vehicle was administered to rats daily after the first paclitaxel injection. Capsaicin was prepared in DMSO as a stock and diluted into Phosphate buffer solution (PBS) (1:1000). Capsaicin was applied via intraplantar injection (3 μg/50 μL) to the dorsal part of the ipsilateral hind paw after each EA treatment. Rats of the control group were administered with vehicle (0.1% DMSO in PBS) only.

### 4.7. Immunofluorescence Staining

Rats were sacrificed on day 14. Rats were deeply anesthetized with sodium pentobarbital (40 mg/kg, i.p.) and perfused through the ascending aorta with 0.9% saline (4 °C) followed by 4% paraformaldehyde in 0.1 M PBS. The L4–6 dorsal root ganglia (DRGs) and the spinal cord were removed, fixed in 4% paraformaldehyde for 6 h, and then cryo-protected in 30% sucrose solution. Transverse spinal cord sections (25 µm) and longitudinal DRG sections (10 µm) were cut on a frozen microtome (Thermo NX50, MA, USA), mounted on gelatin-coated glass slides as 8 sets of every 5th serial section, and processed for immunofluorescent staining. After blocking in 5% normal donkey serum in Tris buffered saline tween (TBST) for 1 h at 37 °C, they were incubated overnight with corresponding primary antibodies. The primary antibodies used were rabbit anti-TRPV1 (ab6166, Abcam), mouse anti-GFAP (c9205, Sigam), mouse anti-OX42 (ab1211, Abcam), mouse anti-NeuN (ab177487, Abcam). and rabbit anti-NeuN (ab177487, Abcam). After washing, the sections were then incubated with Cy3-, Cy5-. or Fluorescein isothiocyanate (FITC)-conjugated secondary antibodies for 1 h at 37 °C. Sections were viewed by Nikon A1R laser scanning confocal microscope (Nikon, Japan). For image quantification, uniform microscope settings were maintained throughout all image capture sessions and experimenters were blinded to treatment groups. For quantification of the % of TRPV1 positively stained DRG neurons, the number of TRPV1 positively stained DRG neurons were divided by the total number of DRG neurons identified by positive NeuN staining; 3–5 images were randomly selected per rat tissue, averaged, and then compared according to methods described in our previous studies [56,57].

### 4.8. Western Blotting

Rats were sacrificed after EA treatment and behavioral test on day 14. Rats were deeply anesthetized with sodium pentobarbital (Nembutal, 40 mg/kg, i.p.) and perfused through the ascending aorta with 0.9% saline (4°C). Then, the L4–6 segments of the DRG were rapidly removed on ice. Tissues were immediately removed and stored at −80 °C. Tissues were homogenized in Radio-Immunoprecipitation assay (RIPA) buffer (50 mM Tris (pH 7.4), 150 mM NaCl, 1% Triton X-100, 1% sodium deoxycholate, sodium orthovanadate, 0.1% Sodium dodecyl sulfonate (SDS), Ethylene diamine tetraacetic acid (EDTA), sodium fluoride, leupeptin, and 1 nM PMSF). The homogenate was allowed to rest on ice for 30 min and was then centrifuged at 15,000 rpm for 15 min at 4 °C, and the supernatant was collected. The protein concentration was determined using the Bicinchoninic acid (BCA) method according to the kit’s instruction (Thermo Fisher, USA), and 10 μg of protein was loaded in each lane. Protein samples were separated on 5–10% SDS-PAGE gels and electrophoretically transferred to polyvinyl difluoride (PVDF) membranes (Bio-Rad, United States). The membranes were blocked with 5% non-fat milk in Tris-buffered saline (TBS) with 0.1% Tween-20 (pH 7.5) at room temperature for 1 h, followed by overnight incubation at 4 °C with the following primary antibodies diluted in blocking buffer: mouse anti-TLR4 (sc-293072, Santa Cruz Biotechnology), rabbit anti-MyD88(CST-4283, CST), and rabbit anti-TRPV1 (ACC-030, Alomone Labs). Subsequently, the immunoblots were incubated with the 2nd antibodies for 2 h at room temperature. Mouse anti-beta actin-loading control (HRP) (ab20272, Abcam) was used as internal control. The immunoreactivity was detected using enhanced chemiluminescence (BIO-RAD, USA) and visualized with an Image Quant LAS 4000 (GE, USA). The density of each band was measured using Image Quant TL 7.0 analysis software (GE, USA). The mean expression level of the target protein in the animals in the vehicle group was considered to be 100%, and the relative expression level of the target protein in all animals was adjusted as a ratio to the level of the vehicle group.

### 4.9. Cell Culture

Rats were sacrificed on day 14. L4–6 dorsal root ganglia (DRGs) were harvested and dissociated using collagenase type 1 and dispase (Gibco, USA) as described previously [58,59]. DRG neurons were cultured in Dulbecco’s modified eagle medium (DMEM) plus 10% fetal bovine serum on round coverslips coated with poly-D-lysine (Sigma, MO, USA) and mouse laminin (Invitrogen, CA, USA) in a 24-well chamber.

### 4.10. Ca^2+^ Imaging

DRG neurons were used 4 h after dissociation. Cells were loaded with Fura 2-AM (10 μM, Invitrogen) for 45 min in a loading buffer containing: NaCl 140, KCl 5, CaCl_2_ 2, MgCl_2_ 2, HEPES 10 (pH 7.4 adjusted with NaOH). Cells were subsequently washed three times and imaged in the loading buffer. Ratiometric Ca^2+^ imaging was performed on a Nikon ECLIPSE Ti-S (Japan) microscope with a Polychrome V monochromator (Till Photonics) and an Orca Flash 4.0 CCD (Charge coupled device) camera (Hamamatsu, Japan). Images were captured and processed with MetaFluor software (Molecular Devices, CA, USA). Ratiometric images were obtained with exposures of 0.5 ms at 340 nm and 0.3 ms at 380 nm excitation wavelengths. Representative Ca^2+^ imaging images were generated using ImageJ software. A cell was considered responsive if the peak Ca^2+^ response was above 20% of the baseline, according to our previous publications [60,61].

### 4.11. Statistical Analysis

Statistical analysis was performed with SPSS 19.0 (Statistic package for social science)(SPSS Inc., Chicago, USA). Results were expressed as mean ± SEM. One-way or two-way ANOVA followed with Tukey’s post hoc test was used for comparison among groups ≥ 3. Comparison was considered significantly different if *p* < 0.05.

## 5. Conclusions

Our study demonstrates that EA treatment ameliorates pain hypersensitivities in a rat model of paclitaxel-induced neuropathic pain. The mechanistic studies reveal that EA suppresses TLR4 and its downstream signaling molecule MyD88 overexpression in DRGs of paclitaxel-treated rats. EA reduces TRPV1 overexpression and channel functional activity in DRG neurons and further suppresses spinal glial cell activation from paclitaxel-treated rats. Our work supports EA as a potential therapeutic option for paclitaxel-induced neuropathic pain in clinic.
